# *UNC13A* Polymorphism Influences Survival in Patients with Frontotemporal Dementia

**DOI:** 10.1002/ana.27245

**Published:** 2025-04-11

**Authors:** Lianne M. Reus, Sean W. Willemse, Sterre C.M. de Boer, Julie De Houwer, Willem L. Hartog, Merel O. Mol, Jeroen G. J. van Rooij, Niccolo Tesi, Laura Donker Kaat, Marc Hulsman, Everard G. B. Vijverberg, Henne Holstege, Wouter van Rheenen, Jan H. Veldink, Leonard H. van den Berg, John C. van Swieten, H. Seelaar, Sven J. van der Lee, Michael A. van Es, Yolande A. L. Pijnenburg

**Affiliations:** ^1^ Alzheimer Center Amsterdam, Neurology, Vrije Universiteit Amsterdam, Amsterdam UMC Location VUmc Amsterdam The Netherlands; ^2^ Amsterdam Neuroscience, Neurodegeneration Amsterdam The Netherlands; ^3^ Center for Neurobehavioral Genetics, Semel Institute for Neuroscience and Human Behavior, David Geffen School of Medicine University of California Los Angeles CA; ^4^ Department of Neurology UMC Utrecht Brain Center Rudolf Magnus Utrecht The Netherlands; ^5^ Department of Neurology Erasmus Medical Center Rotterdam The Netherlands; ^6^ Department of Clinical Genetics Erasmus Medical Center Rotterdam The Netherlands; ^7^ Genomics of Neurodegenerative Diseases and Aging, Human Genetics Vrije Universiteit Amsterdam, Amsterdam UMC Location VUMC Amsterdam The Netherlands; ^8^ Delft Bioinformatics Lab Delft University of Technology Delft The Netherlands

## Abstract

*UNC13A* (rs12608932‐CC) is associated with both amyotrophic lateral sclerosis (ALS) and frontotemporal dementia (FTD), and shortens survival in ALS. We aim to describe the association for *UNC13A* and survival in FTD. We included 626 patients with FTD from Dutch memory clinics, including a subcohort of 150 patients with TDP‐43 pathology. Survival analyses were performed using Cox proportional hazard models in a recessive manner. Homozygosity for rs12608932‐C in *UNC13A* was associated with a shorter survival compared with other genotypes (hazard ratio [HR] = 1.28, 95% confidence interval [CI] = 1.02–1.60, *p* = 0.033), which has implications for patient counselling and trial design. ANN NEUROL 2025;97:1062–1066

Frontotemporal dementia (FTD) is the second most common young‐onset dementia, characterized by behavioral changes and aphasia as main symptoms.^1^ FTD is part of a disease continuum with amyotrophic lateral sclerosis (ALS), a neurodegenerative disease characterized by motor neuron loss in the spinal cord and brain leading to spasticity, progressive weakness, and respiratory failure. Approximately 5 to 15% of patients with FTD develop motor neuron involvement, whereas 5 to 10% of patients with ALS meet the diagnostic criteria for FTD and nearly half develop cognitive or behavioral deficits in the frontotemporal spectrum.^2^, ^3^

The discovery of TDP‐43 mislocalization and aggregation as a pathological hallmark for both FTD and ALS has explained the relation between these diseases. In FTD, TDP‐43 pathology is present in around 50% of cases, with the other half comprising of Tau pathology or, more rarely, fused‐in sarcoma (FUS).^1^ In ALS, approximately 97% of ALS cases are characterized by TDP‐43 pathology, with only a small percentage (~3%) showing FUS or superoxide dismutase 1 (SOD1) pathology.^2^

Besides shared pathological and clinical characteristics, there is a common genetic basis. Familial forms of ALS and FTD can be caused by rare pathogenic variants in a number of genes, including *C9orf72*, *TBK1*, and *TARDBP*. Genome‐wide association studies have also identified common genetic variants within an intron of *UNC13A* as a shared risk factor. The most significant association in both ALS and FTD is a single nucleotide polymorphism (SNP) at rs12608932. Subsequent studies in ALS have shown that homozygosity for the C‐allele at rs12608932 in *UNC13A* modifies the clinical phenotype, implicating shorter survival and a higher frequency of bulbar‐onset, cognitive impairment and FTD.^4^, ^5^, ^6^

Recently, the pathophysiological mechanism driving this effect has been elucidated. The relevant intron within *UNC13A* contains a cryptic exon, which is incorporated into mRNA under conditions of TDP‐43 pathology.^7^, ^8^ In turn, this leads to nonsense‐mediated decay of the mRNA in these cells, causing a loss of functional UNC13A and profound deficits in synaptic transmission.^9^

Considering *UNC13A* confers risk for both ALS and FTD and the effect on the ALS phenotype is TDP‐43 driven, we hypothesized that homozygosity for the C‐allele at rs12608932 in *UNC13A* negatively impacts survival in TDP‐43 associated FTD.

## Methods

In total, 708 patients with FTD were selected from memory clinic cohorts in the Netherlands, including 290 patients from the Erasmus Medical Center (EMC) in Rotterdam and 418 from the Amsterdam Dementia Cohort (ADC). These cohorts have been described previously.^10^, ^11^ Both have been approved by a Medical Ethical Committee and all included patients or their carers have given informed consent for the use of their clinical and genetic data in scientific research. We collected the following clinical data: sex, age/time at study entry, age/time at onset, survival (defined as the time from first symptoms until censoring or death), date/time of censoring or date/time of death (taken from population registries), and clinical FTD subtype. Clinical subtyping included behavioral variant FTD (bvFTD), semantic variant primary progressive aphasia (svPPA), non‐fluent agrammatic primary progressive aphasia (nfvPPA) and FTD with motor neuron symptoms (FTD‐ALS). For both cohorts, patients were excluded if follow‐up data and/or genetic data (rs12608932 on *UNC13A*) were not available. Patients were genotyped with the Illumina Global Screening Array (GSA) and genotypes of rs12608932 were extracted. Quality control of the samples has been described in depth elsewhere.^12^

As we hypothesized that homozygosity for the C‐allele at rs12608932 in *UNC13A* (henceforth referred to as rs12608932‐CC) has an effect on survival in the presence of TDP43‐pathology, we excluded patients with postmortem pathology other than TDP‐43 and/or Mendelian mutation related to non‐TDP‐43 pathologies (n = 33). For 104 patients, postmortem pathology confirmation was available. The final total cohort included 626 patients. We further established a TDP‐43 subcohort. Patients were classified to the TDP‐43 subcohort if they carried a TDP‐43‐related Mendelian mutation, had TDP‐43 pathology postmortem, or had a clinical subtype indicative of TDP‐43 pathology (ie, FTD‐ALS), resulting in a TDP‐43 subcohort of 150 patients. The details for in‐ and exclusion of patients can be found in Supplementary Figure S1.

We performed survival analysis using Cox proportional hazard models with adjustment for left truncation, correcting for age at onset, sex, and study site. Median survival was calculated using a Kaplan–Meier estimate and presented with a 95% confidence interval (95% CI). We based groups on *UNC13A* genotype in a recessive manner (ie, rs12608932‐AA/AC vs CC). Additionally, we performed multiple sensitivity analyses: excluding Mendelian mutation carriers, stratifying according to clinical subtype, and including only patients with a known TDP‐43 burden. Due to pre‐planned subgroup analysis based on a singular hypothesis, we did not correct for multiple testing. Analysis were performed using R software version 4.3.1. The *p* values lower than 0.05 were considered significant.

## Results

Demographic results of the total sample (N = 626, mean age = 63.2 ± 8.2 years, 43% female patients) are presented in Table 1. The rs12608932‐CC (n = 114) carriers did not differ from the other genotype group (n = 512 A/C and A/A) in terms of age at study entry, age at onset, and number of Mendelian mutation carriers. Comparisons on the 2 cohorts (ADC vs. EMC) are provided in Supplementary Table S1.

**Table 1:** Baseline Statistics for the Total and TDP‐43 Cohort, Stratified for *UNC13A* Genotype

	Total Cohort			*p*	TDP‐43 Cohort			*p*
	All (n = 626)	AA/AC (n = 512)	CC (n = 114)		All (n = 150)	AA/AC (n = 111)	CC (n = 39)	
Male, n (%)	356 (57)	303 (59)	53 (46)	**0.017**	78 (52)	62 (56)	16 (41)	0.16
Age at study entry, yr, mean (SD)	63.2 (8.2)	63.3 (8.1)	62.4 (8.7)	0.28	62 (7.6)	62.1 (7.9)	61.6 (7.0)	0.71
Age at onset, yr, mean (SD)	59.6 (8.4)	59.6 (8.3)	59.4 (8.7)	0.81	58.4 (7.5)	58.2 (7.8)	59.1 (7.0)	0.51
Mendelian mutation, n (%)	87 (14)	70 (14)	17 (15)	0.84	87 (58)	70 (63)	17 (44)	0.053
Died, n (%)	488 (78)	394 (77)	94 (82)	0.25	136 (91)	99 (89)	37 (95)	0.47
Survival since onset, mo, median [95% CI]	102 [72.0–141.3]	105.5 [76.6–145.4]	87.8 [54.5–126.2]	**0.001**	85.2 [52.5–119.3]	90 [68.4–123.0]	52.4 [33.9–88.7]	**0.0002**

CI = confidence interval, n = number, SD = standard deviation, TDP‐43 = TAR DNA‐binding protein 43kD.

**Figure 1:**
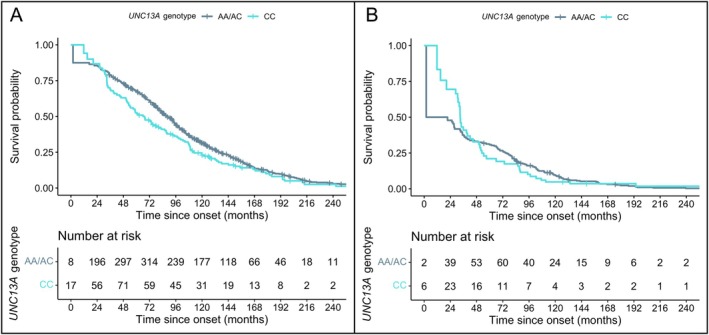
Survival of patients with FTD and patients with TDP‐43 pathology. Panel A shows overall survival of the total cohort, split for homozygosity for the C‐allele at rs12608932 in *UNC13A* in a recessive manner. Panel B shows overall survival of the TDP‐43 cohort, split for homozygosity for the C‐allele at rs12608932 in *UNC13A* in a recessive manner. The number at risk initially increases due to left truncation, where some individuals enter the study at later time points rather than at the onset. FTD = frontotemporal dementia. [Color figure can be viewed at www.annalsofneurology.org]

During 325.8 months of follow‐up, 488 patients (80%) died. The median survival for the total cohort was 102 months (95% CI = 72.0–141.3). For rs12608932‐CC, the median survival was 87.8 months (95% CI = 54.5–126.2) compared to 105.5 months (95% CI = 76.6–145.4) for other genotypes. The rs12608932CC was associated with a shorter survival when compared with other genotypes (hazard ratio [HR] = 1.28, 95% CI = 1.02–1.60, *p* = 0.033; see the Figure 1). The effect remained significant after exclusion of Mendelian mutation carriers (HR = 1.29, 95% CI = 1.01–1.65, *p* = 0.041; Table 2). In the TDP‐43 subcohort (n = 150), we also observed a shorter survival for rs12608932‐CC in comparison with other genotypes (median survival = 52.4 vs 90 months, HR = 1.52, 95% CI = 1.03–2.26, *p* = 0.036). After stratifying for clinical subtype, we only found a difference in survival for rs12608932‐CC carriers versus the other genotypes for bvFTD (HR = 1.38, 95% CI = 1.03–1.84, *p* = 0.03). Further results stratified for clinical and pathological subtype are shown in Table 2.

**Table 2:** Cox Proportional Hazard Models for Survival for Patients With FTD Homozygous for the C‐allele at rs12608932 in *UNC13A* in a Recessive Manner, Split for Clinical, and Pathological Subtype

	N	HR [95% CI]	*p*
Total cohort	626	1.28 [1.02–1.60]	**0.033**
Total excluding Mendelian mutations	539	1.29 [1.01–1.65]	**0.041**
*C9ORF72* carriers	59	0.99 [0.41–2.37]	0.97
Clinical subtype			
bvFTD	350	1.38 [1.03–1.84]	**0.030**
svPPA	104	1.11 [0.5–2.49]	0.79
nfvPPA	54	0.82 [0.4–1.69]	0.60
FTD‐ALS	36	1.43 [0.67–3.03]	0.35
Pathological subtype			
TDP‐43	150	1.52 [1.03–2.26]	**0.036**
Tau	32	1.58 [0.42–6.0]	0.50

bvFTD = behavioral variant frontotemporal dementia, CI = confidence interval, FTD‐ALS = frontotemporal dementia with motor neuron symptoms, HR = hazard ratio, nfvPPA = non‐fluent agrammatic primary progressive aphasia, svPPA = semantic variant primary progressive aphasia, TDP‐43 = TAR DNA‐binding protein 43kD.

## Discussion

In line with previous observations in ALS, we found a shorter survival of 17.7 months for patients with FTD homozygous for the C‐allele at rs12608932 in *UNC13A* in comparison to AA/AC‐genotypes. We were able to confirm this finding across multiple sensitivity analyses, showing the effect is independent of Mendelian mutations. Although the TDP‐43 cohort was smaller, the effect seemed stronger when compared with the total cohort with a survival difference of more than 35 months, which might support our assumption that the negative effect on survival is TDP‐43 dependent. Unfortunately, the number of patients with certain Tau‐associated FTD was limited and we are therefore unable to draw firm conclusions on whether the effect *UNC13A* has on survival is indeed absent under conditions of Tau pathology.

As 1 in 6 people is homozygous for rs12608932‐C, our findings yield implications for a large number of patients with FTD. Knowledge of *UNC13A* genotype holds prognostic value in clinical practice and it seems a factor of relevance, as survival in rs12608932‐CC is shorter compared to that previously described for genetic mutations.^13^ This seems especially true in the case of TDP‐43 pathology, with a difference in survival of more than 3 years. At present, discrimination between FTD with TDP‐43 or Tau pathology depends largely on autopsies. A recent finding that plasma derived extracellular vesicles can be used as biomarkers for TDP‐43 or Tau pathology would allow application of this knowledge while patients are alive.^14^ Furthermore, *UNC13A* genotype could be a factor to correct or stratify for in clinical trials in order to prevent skewed survival in a trial arm.

In addition, our findings further strengthen the commonality of FTD and ALS. The rs12608932‐CC in *UNC13A* is associated with a higher incidence of cognitive deficits and concomitant diagnosis of FTD when patients are diagnosed with ALS.^6^, ^9^ Additionally, patients with ALS with rs12608932‐CC have more cortical atrophy on magnetic resonance imaging (MRI) and more hypometabolism on positron emission tomography (PET) in the frontal and temporal lobes, when compared with other genotypes.^6^, ^15^ Further determination of the *UNC13A* phenotype in FTD is necessary in order to assess whether these findings are unique to rs12608932‐CC in ALS or are also present in such patients with FTD.

In summary, we find that homozygosity for the risk polymorphism at rs12608932 in *UNC13A* leads to shorter survival in patients with FTD. Future research elucidating the clinical phenotype of *UNC13A* in FTD and its similarities to ALS could warrant collective efforts for the development of *UNC13A*‐specific treatments.

## Author Contributions

L.M.R., S.W.W., S.J.vdL., W.vR., J.H.V., L.H.vdB., M.A.vE., and Y.A.L.P. contributed to conception and design of the study; L.M.R., S.C.M.dB., J.D.H., W.L.H., M.O.M., J.G.J.vR., N.T., L.D.K., M.H., H.H., J.vC., H.S., S.J.vdL., and Y.A.L.P. contributed to the acquisition and analysis of data; L.M.R. and S.W.W. contributed to drafting the text or preparing the figures.

## Potential Conflicts of Interest

Nothing to report.

## Supporting information


**Supplementary Figure S1:** Flowchart with patient in‐ and exclusion for the total cohort and the TDP‐43 cohort. Panel A shows patient in‐ and exclusion for the total cohort. Panel B shows patient in‐ and exclusion for the TDP‐43 cohort.

**Supplementary Table S1:** Baseline statistics stratified for recruitment site.

## Data Availability

Data can be made available upon request. Data sharing of subject‐level genetic and clinical data may be restricted by consent given by research participants within each contributing cohort and by European GDPR regulations, which currently excludes data sharing with a number of non‐European countries.
