# Supplementary material for: UNC13A Polymorphism Influences Survival in Patients with Frontotemporal Dementia
Source: Ann Neurol. 2025 Apr 11;97(6):1062–6. doi: 10.1002/ana.27245 (PMC12082005; doi:10.1002/ana.27245)
Supplement: Supplementary file 2 — Supplementary Table S1: Baseline statistics stratified for recruitment site. [file ANA-97-1062-s001.docx]

**Supplementary table 1: Baseline statistics stratified for recruitment site.**

|  | **Cohort** | | |  |
| --- | --- | --- | --- | --- |
|  | Total  (n = 626) | ADC  (n = 392) | EMC  (n = 234) | *p* |
| Male, n (%) | 356 (57) | 236 (60) | 120 (51 | **0.035** |
| Age at study entry, years, mean (SD) | 63.2 (8.2) | 63.8 (7.8) | 62.1 (8.8) | **0.011** |
| Age at onset, years, mean (SD) | 59.6 (8.4) | 60.5 (8.2) | 58.1 (8.4) | **0.0005** |
| Mendelian mutation, n (%) | 87 (14) | 43 (11) | 44 (19) | **0.0087** |
| Died, n (%) | 488 (78) | 258 (66) | 230 (98) | **6.4x10^-21^** |
| Survival since onset, months, median [95% CI] | 102 [72.0-141.3] | 101.7 [70.12-135.6] | 103.8 [73.5-149.7] | 0.27 |

*ADC = Amsterdam Dementia Cohort, CI = Confidence Interval, EMC = Erasmus Medical Center, n = number, SD = standard deviation, TDP-43 = TAR DNA-binding Protein 43kD.*
